# Correlation Between Co Levels in Hair and Blood of Patients Who Underwent Metal-on-metal Hip Arthroplasty

**DOI:** 10.1016/j.artd.2022.09.006

**Published:** 2022-10-13

**Authors:** Marco Di Luzio, Anna Ronchi, Marilina Amabile, Enrico Tassinari, Massimo Oddone, Giancarlo D’Agostino

**Affiliations:** a Istituto Nazionale di Ricerca Metrologica (INRIM), Pavia, Italy; b Laboratorio di Tossicologia Clinica e Sperimentale, Centro Antiveleni di Pavia - Centro Nazionale di Informazione Tossicologica, Istituti Clinici Sientifici Maugeri IRCCS Pavia, Pavia, Italy; c Laboratorio di Tecnologia Medica, IRCCS Istituto Ortopedico Rizzoli, Bologna, Italy; d Ortopedia-Traumatologia e Chirurgia protesica e dei reimpianti d'anca e di ginocchio, IRCCS Istituto Ortopedico Rizzoli, Bologna, Italy; e Dipartimento di Chimica, Università di Pavia, Pavia, Italy

**Keywords:** Hair analysis, Metal-on-metal, MoM hip revision, Cobalt

## Abstract

**Background:**

The purpose of this paper is to study the dependence of Co levels in hair on Co levels in blood after metal-on-metal (MoM) hip replacement and prove the suitability of hair analysis coupled to blood analysis in the decision process regarding implant revision evaluation.

**Methods:**

Hair samples of 19 MoM patients having both well-functioning and malfunctioning implants and Co mass concentration levels in blood between 0.2 μg L^-1^ and 221.0 μg L^-1^ were included. A method based on inductively coupled plasma mass spectrometry was validated and used to measure the Co level in hair.

**Results:**

The Co mass fraction in the hair of patients ranged between 0.011 mg kg^-1^ and 0.712 mg kg^-1^. A correlation analysis showed a statistically significant positive correlation (*r* = 0.932, *P* < .001) between Co in the hair and that in the blood in the full-level range and a statistically nonsignificant positive correlation (*r* = 0.595, *P* = .091) in the low-level range.

**Conclusions:**

A correlation between the Co level in the hair and that in the blood exists when the latter is clearly above the 7 μg L^-1^ mass concentration threshold suggested for implant revision evaluation. The correlation disappears when the Co level in blood approaches or falls down the mass concentration threshold and that in the hair approaches or falls within the normal population range of 0.004-0.14 mg kg^-1^. Accordingly, clinicians could consider a hair analysis coupled to a blood analysis to assess the revision of malfunctioning MoM implants that release metals in patient’s body.

## Introduction

Since the early years of hip arthroplasty, metal-on-metal (MoM) bearings have been used as a possible alternative to metal-on-polyethylene bearings, both in total hip replacements (THRs) and hip resurfacings (HRs).

Although metal-on-polyethylene bearings have been preferred in most cases over the MoM ones, a new generation of MoM bearings has been developed and extensively implanted starting from the late 1990s, taking advantage of the availability of better materials made from Co-Cr alloys and manufactured with improved hardening methods [1].

At first, the new generation of MoM bearings showed good outcomes, especially in young patients requiring enhanced mechanical performance [2]. However, later clinical studies pointed out higher revision rates and manifestation of adverse reactions and pseudo-tumors due to small metallic particles and ions produced by wear and corrosion [3], the latter being particularly relevant in taper junctions of large-head MoM-THR implants [4].

Following the acknowledgment of these findings, health organizations have advised against the use of MoM bearings [5]. Despite the dramatic decline in the current use of MoM bearings in hip arthroplasty, a large number of patients have been treated with MoM-THRs and MoM-HRs [6], and the need for close monitoring of these patients is commonly acknowledged.

Most up-to-date guidelines on patient assessment and management as well as a compendium of adverse reactions to metal debris have been reviewed by Derummond et al. [7]. The reported guidelines are based on the final opinion statement on MoM hip arthroplasty published by the Scientific Committee on Emerging and Newly Identified Health Risks [8]. Imaging and blood metal (Co, Cr) ion testing are recommended, and follow-up time frames are outlined both for asymptomatic and symptomatic patients. Implant revision evaluation is recommended depending on imaging abnormalities and blood Co ion levels rising above the 7 μg L^-1^ mass concentration threshold.

Hair metal testing is also suggested by the Scientific Committee on Emerging and Newly Identified Health Risks as a diagnostic tool that may prove useful in the decision-making process regarding the implant revision. To date, there are very few published studies showing the suitability of hair analysis in this framework. Fu-Cun et al. [9] reported in 2010 a significant increase of Co and Cr levels in the hair of a patient group after an MoM implant surgery and concluded that monitoring the observed increase might be a fit-for-purpose indication of prostheses failure. de la Flor at al. published in 2013 a comparative study [10] of concordance tests between Co and Cr levels in serum, urine, and hair and concluded that hair is a good biologic marker for monitoring metals released from MoM prostheses and might have clinical application in patients’ follow-up.

Although findings of these studies are promising, in fact, hair analysis has not been adopted in implant revision evaluation, albeit hair collection and storage are less invasive, faster, simpler, and less expensive than blood collection, and quantification is feasible using routine analytical methods with costs similar to those adopted in blood analysis.

Aiming at filling in the current lack of knowledge on hair analysis as a diagnostic tool in MoM patients’ assessment, we carried out a further study to investigate the existence of a correlation between metal levels in hair and in blood. We selected Co as a target element because we have previously proved that the hair washing protocol proposed by the International Atomic Energy Agency (IAEA) is suitable for Co determinations in hair [11].

## Material and methods

Institutional review board approval for the present study was obtained by the Bioethics Committee of Area Vasta Emilia Romagna Centro (protocol no. 9103, 30th July 2018). From March to December 2018, we assessed for eligibility of 125 MoM patients during their follow-up control. We collected hair samples of approximately 2 cm length from the nape of the neck of 73 patients after excluding 52 patients with colored or treated hair. Cleaned ceramic scissors were used to limit additional metal contamination. Hair samples were packed in polyethylene bags and stored at the ambient temperature.

As required by the follow-up protocol, blood was collected following the precautions for trace metal elements analysis: Samples were obtained using a disposable butterfly needle; the first 3 mL was discarded, and further 7 mL of blood was withdrawn and transferred into a separate trace-element BD Vacutainer (BD, Franklin Lakes, NJ) tube containing ethylenediaminetetraacetic acid. The determination of Co mass concentration in blood samples, *c*_Co blood_, was carried out by routine laboratory atomic absorption spectrometry measurements at the Laboratorio Unico Metropolitano of Bologna. Co levels in blood measured in previous controls of some of the selected patients and in measurements carried out since July 2017 were available.

Based on the results of the blood analysis, we divided the patients in 2 groups: The first (group A) consisted of 59 patients with *c*_Co blood_ between 0.1 μg L^-1^ and 7 μg L^-1^, and the latter (group B) consisted of 14 patients with *c*_Co blood_ between 7 μg L^-1^ and 221.0 μg L^-1^. Since patients in group A are expected to have low amounts of Co released from the MoM prosthesis, we excluded 43 patients whose hair sample mass was less than 250 mg to limit the measurement uncertainty due to homogeneity.

The collected hair samples of the 30 selected patients were washed according to the IAEA protocol to eliminate the unbound Co content; hereafter, the measured Co mass fraction value in hair, *w*_Co hair_, is the bound component. We settled and used an automatic washing system to assure maximum reproducibility of the procedure and to limit labor time.

After air-drying at room temperature, samples were weighted and placed in cleaned polyethylene containers and sealed; mass determinations were performed using a calibrated analytical balance. At the same time, we determined the moisture correction factor by thermobalance using additional hair samples subjected to the same washing and air-drying cycle.

Hair analyses were performed at the Laboratorio di Tossicologia Clinica e Sperimentale (LTCS) of the Centro Antiveleni of Pavia and Centro Nazionale Informazioni Tossicologiche, Istituti Clinici Scientifici Maugeri, (IRCCS) Pavia, using a method based on inductively coupled plasma mass spectrometry (ICP-MS). Samples were removed from their containers, acid-digested in a microwave oven, and stored at room temperature until analysis.

Co determinations were carried out using a mass spectrometer (ELAN 6100 DRC II ICP-MS; Perkin-Elmer SCIEX Instruments, Ontario, Canada) equipped with a cyclonic spray chamber and Meinhard-type concentric nebulizer, 2 interface cones (sampler and skimmer), a quadrupole mass filter, and a dynamic reaction cell. This mass spectrometer allows obtaining background values <1 cps and instrumental detection limits in the order of ng L^-1^. Based on the results of the blood analysis, we excluded 10 patients of group A and 1 patient of group B because Co in their hair was below the measurement detection limit.

**Figure 1:**
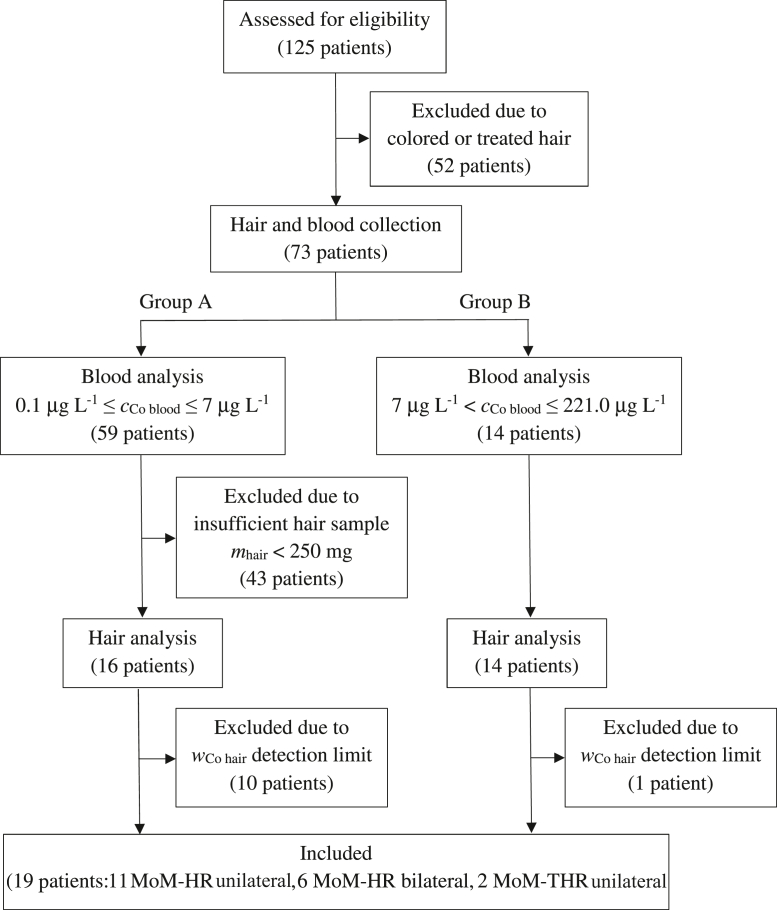
Flowchart summarizing the selection of patients included in this study.

In conclusion, 19 patients were included in this study, 7 females (age 63.7 ± 18.4 years, range 33-80 years) and 12 males (age 62.4 ± 11.4 years, range 43-80 years) having both well-functioning and malfunctioning implants. At the time of hair sampling, 17 patients had MoM-HR arthroplasty (11 unilateral and 6 bilateral), and 2 patients had MoM-THR arthroplasty (both unilateral). The flowchart summarizing the selection of patients is shown in Figure 1.

To assure the confidence in the quality of Co determinations performed at the LTCS, we preliminarily carried out an intercomparison between the LTCS and the Istituto Nazionale di Ricerca Metrologica (INRIM). A fit-for-purpose primary ratio method described by D’Agostino et al. [11] and based on the instrumental neutron activation analysis (INAA) was applied by the INRIM to establish direct traceability of the results.

The intercomparison was performed using hair samples of a healthy person without a joint prosthesis and samples of the human hair reference material ERM-DB001 (SigmaAldrich, St. Louis, MO) [12].

About 4 g of hair of the healthy person were cut to 2 cm length, washed according to the IAEA protocol, homogenized, and divided into 8 samples of 500 mg; 5 samples were measured by the LTCS using the ICP-MS method adopted in this study, and 3 samples were measured by the INRIM using the INAA primary ratio method. In addition, both laboratories measured a 500-mg sample of the ERM-DB001.

## Results

**Figure 2:**
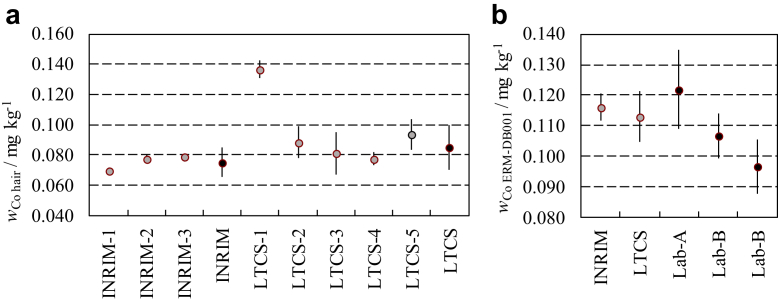
Co mass fraction values measured in hair samples of a healthy person (a) and of the ERM-DB001 (b). Gray and black dots represent single and average results, respectively. Error bars, if visible, indicate an expanded uncertainty (k = 2) and refer to the single measurement uncertainty or to the experimental standard deviation in the case of single or average results, respectively. The LTCS average value does not include the LTCS-1 value. INRIM, Istituto Nazionale di Ricerca Metrologica; LTCS, Laboratorio di Tossicologia Clinica e Sperimentale.

Results of the intercomparison carried out by the INRIM and LTCS are shown in Figure 2. Co mass fraction values in the hair of the healthy person (Fig. 2a) are within the Co reference range of normal population, 0.004-0.14 mg kg^-1^, proposed by Goullé et al. [13] and in agreement with the results we previously obtained with the same person, that is, the control subject C2 reported by D’Agostino et al. [11]. Conversely, the 4.3 mg kg^-1^ preoperation Co mass fraction mean value reported by Fu-Cun et al. [9] is remarkably above the upper limit of the normal population reference range.

The same applies to the results obtained with the ERM-DB001, a material produced starting from uncolored and untreated human hair. The certificate report gives 0.106 mg kg^-1^ mass fraction as the informative value of Co based on results provided by 2 laboratories, here called Lab-A and Lab-B. The first laboratory used INAA to collect 1 data set, and the latter used ICP-MS to collect 2 data sets. Values are shown in Figure 2b.

**Figure 3:**
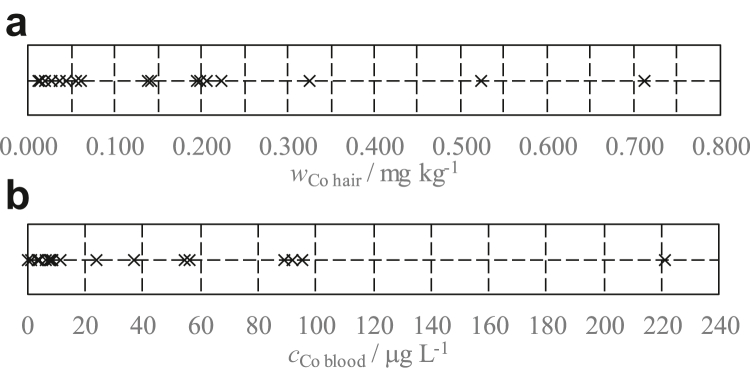
Co mass fraction values measured in hair samples (a) and Co concentration values measured in blood samples (b) of the 19 MoM patients. MoM, Metal-on-metal

The outcomes of hair and blood analyses of 19 MoM patients performed by the LTCS and the Laboratorio Unico Metropolitano, respectively, are graphically shown in Figure 3a and b, respectively, and listed in Table 1.

**Table 1:** Blood Co concentration, *c*_Co blood_, and hair Co mass fraction, *w*_Co hair_, measured in samples of 19 MoM patients.

Patient	Implant	*c*_Co blood_/μgL^-1^	*w*_Co hair_/mgkg^-1^
P1	MoM-HR bilateral∗	221.0	0.712 (115)
P2	MoM-HR unilateral∗	95.3	0.193 (34)
P3	MoM-THR unilateral∗	92.0	0.523 (91)
P4	MoM-HR bilateral∗	89.1	0.325 (64)
P5	MoM-HR unilateral	56.0	0.198 (34)
P6	MoM-HR bilateral	54.3	0.206 (55)
P7	MoM-HR unilateral	37.0	0.222 (46)
P8	MoM-HR unilateral	24.0	0.043 (7)
P9	MoM-HR unilateral∗	11.7	0.142 (23)
P10	MoM-HR unilateral∗	11.2	0.138 (24)
P11	MoM-HR unilateral	8.5	0.043 (8)
P12	MoM-HR bilateral	7.9	0.054 (9)
P13	MoM-HR bilateral	7.4	0.036 (7)
P14	MoM-HR unilateral	6.5	0.035 (8)
P15	MoM-HR unilateral	4.3	0.059 (11)
P16	MoM-THR unilateral	4.2	0.013 (2)
P17	MoM-HR bilateral	3.4	0.018 (4)
P18	MoM-HR unilateral	1.0	0.011 (2)
P19	MoM-HR unilateral	0.2	0.027 (5)

The implant type is specified, and patients with malfunctioning MoM bearings who have undergone a ceramic-on-ceramic (CoC) revision surgery are indicated with an asterisk.

The brackets refer to the expanded uncertainty (k = 2) and apply to the last digits. MoM-HR, Metal-on-metal hip replacement

Based on the results of hair measurements, 2 distinct subgroups are identified: one shrunk between 0.011 mg kg^-1^ and 0.059 mg kg^-1^ (10 patients), and the other extended between 0.138 mg kg^-1^ and 0.712 mg kg^-1^ (9 patients). A similar situation occurs with subgroups identified by blood measurements: one shrunk between 0.2 μg L^-1^ and 11.7 μg L^-1^ (11 patients), and the other extended between 24.0 μg L^-1^ and 221.0 μg L^-1^ (8 patients). There is a good agreement between the division of patients into subgroups according to Co values in blood and hair. If we removed P8, P9, and P10, the agreement would be 100%.

**Figure 4:**
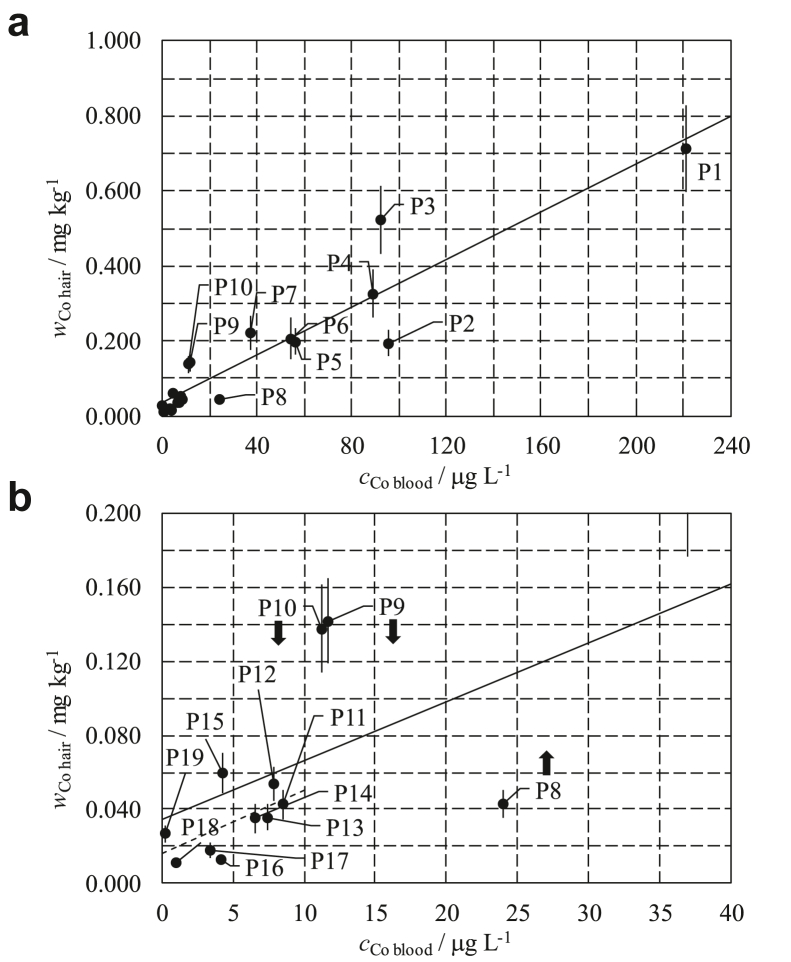
Hair Co mass fraction, *w*_Co hair_, vs blood Co concentration, *c*_Co blood_, measured in samples of 19 MoM patients, labeled P1-P19. Full-level range (a) and a zoom of the low-level range (b) are shown. The solid straight line is fitted to the P1-P19 data set while the dotted straight line is fitted to the P11-P19 data set. Error bars indicate the expanded uncertainty (k = 2), and arrows point out expected Co level variations in hair according to patient’s history.

To observe the correlation between hair and blood Co levels, *w*_Co hair_ values are plotted vs *c*_Co blood_ values in Figure 4 and labeled with the patient number identification assigned in Table 1. The solid straight line in Figure 4a and b is fitted to the full-level range data (patients P1-P19), while the dotted straight line in Figure 4b is fitted to the low-level range data (patients P11-P19).

It is worth noting that Co levels in hair measured in this study are significantly lower than those quantified by Fu-Cun et al. [9] in MoM patients having well-functioning implants and by de la Flor et al. [10] in MoM patients having both well-functioning and malfunctioning implants. In detail, the 61.98 mg kg^-1^ and 53.28 mg kg^-1^ mean values reported in the studies by Fu-Cun et al. and de la Flor et al. [9,10], respectively, are 2 order of levels higher than the 0.712 mg kg^-1^ of patient P1 albeit Co in the patinet’s blood is 31-fold the 7 μg L^-1^ mass concentration threshold.

## Discussion

The quality and metrological traceability of hair analysis carried out in this study are supported by the use of the IAEA washing method, demonstrated to be suitable to remove the unbound Co component [11], and the agreement between the LTCS and INRIM intercomparison results plotted in Figure 2. In addition, particular attention was paid to the exclusion of hair samples with insufficient mass and results below the measurement detection limit.

A correlation analysis showed a statistically significant positive correlation between *w*_Co hair_ and *c*_Co blood_ (*r* = 0.932, *P* value < .001) if the full-level range data (patients P1-P19) are considered and a statistically nonsignificant positive correlation (*r* = 0.595, *P* value = .091) if only the low-level range data (patients P11-P19) is considered.

This quantitative outcome is in agreement with the qualitative graphical representation shown in Figure 4. P1-P19 data are, to some extent, grouped in a linear shape within the 0-0.800 mg kg^-1^ vs 0-240 μg L^-1^ full-level region, whereas the P11-P19 data are randomly scattered within the 0-0.060 mg kg^-1^ vs 0-10 μg L^-1^ low-level region.

As reported in Table 1, in the low-level region, patients had Co in hair within the 0.004-0.14 mg kg^-1^ normal population reference range [13] and, except for P8, P9 and P10, Co in blood near or below the 7 μg L^-1^ mass concentration threshold. In the high-level region, conversely, patients had Co in hair clearly above the 0.14 mg kg^-1^ upper limit of the normal population reference range and Co in blood definitely above the 7 μg L^-1^ threshold.

A possible reason underlying the anomaly of P8, P9, and P10 and perhaps affecting, to some extent, other data is that the bound Co content of a hair sample reflects the average level of Co circulated in the body during the hair-growing period. Instead, a blood sample reflects the present-day level of Co, which may also be increased from baseline as a result of patients' physical activity [14]. As the hair growth is about 1 cm per month, *w*_Co hair_ values in Figure 4 represent 2 months’ average before hair sampling while *c*_Co blood_ values represent those on the day of hair sampling. In particular, Co values in the blood of P8, P9, and P10 at 3, 4, and 5 months before the day of hair sampling were 7.2 μg L^-1^, 71.5 μg L^-1^, and 55.0 μg L^-1^ compared to 24.0 μg L^-1^, 11.7 μg L^-1^, and 11.2 μg L^-1^ at the day of sampling, respectively. Accordingly, we might expect an upcoming increase in the Co level in the hair of P8 and a decrease in the Co level in the hair of P9 and P10, as indicated by the upward or downward arrows in Figure 4b.

Apart from 6 patients (P1, P2, P3, P4, P9, and P10), the remaining 13 patients had well-functioning MoM implants. P1, P2, P3, P4, and P9 underwent a ceramic-on-ceramic revision surgery after hair sampling while P10 and P19 underwent a ceramic-on-ceramic revision surgery 3 and 1 months before, respectively. The knowledge of the noticeably high Co levels in the hair of patients P1, P3, and P4 would surely help the implant revision evaluation. In addition, monitoring Co levels in the hair of patients P2, P10, P5, P6, and P7 would have been useful in their follow-ups. Patient P19 is a separate case because implant revision was performed for aseptic lymphocyte-dominated vasculitis-associated lesions and not for Co released by the MoM prosthesis.

Major limitations of our study are the small number of involved patients and the lack of repeated blood measurements in the period prior to the hair sampling. On the other hand, reported Co mass fractions obtained also at low-level values in hair are reliable because we used the recommended IAEA hair washing protocol and an ICP-MS method validated by a primary ratio INAA method.

The random scattering of the data in the low-level region points out that establishing a linear relationship between *w*_Co hair_ and *c*_Co blood_ when the latter is below or close to the 7 μg L^-1^ mass concentration threshold is not realistic. The amount of Co in hair due to MoM implants in this region is buried within the Co level in the hair of normal population. Besides, it is quite evident that in the high-level region, that is, when *c*_Co blood_ is significantly above the 7 μg L^-1^ mass concentration threshold, establishing a linear relationship between *w*_Co hair_ and *c*_Co blood_ appears possible, but the average of repeated measurements of Co in blood is required to limit the effect of present-day variations and quantify the patient’s baseline value. In this respect, a further study should be performed with a larger number of patients having Co values in blood regularly monitored in the months preceding the day of hair sampling.

## Conclusions

In conclusion, a correlation between the Co level in hair and that in blood exists when the latter is clearly above the threshold suggested for implant revision evaluation. The correlation disappears when the Co level in blood approaches or falls down the threshold and that in hair approaches or falls within the normal population range. Accordingly, hair is confirmed as a suitable biomarker for Co released by severe failures of MoM implants.

Clinicians should consider hair analysis in addition to blood analysis in order to evaluate the hip implant revision of a patient. Establishing a Co threshold level in hair corresponding to the already available Co threshold level appears to be challenging. However, exceeding the Co level of 0.20 mg kg^-1^ in hair, that is, about 1.5 times the upper limit of the normal population reference range, when Co in blood is above the 7 μg L^-1^ threshold, should be considered as an additional warning of possible failure of the MoM implant.

## Acknowledgment

The authors are much indebted to Dr. Susanna Stea for collaborating in the realization of the study and grateful to Stefania Ramirez for providing technical support and valuable information.

## Conflicts of interest

The authors declare there are no conflicts of interest.

For full disclosure statements refer to https://doi.org/10.1016/j.artd.2022.09.006.

## Appendix A Supplementary data

Conflict of Interest Statement for Amabile

Conflict of Interest Statement for D’Agostino

Conflict of Interest Statement for Di Luzio

Conflict of Interest Statement for Oddone

Conflict of Interest Statement for Ronchi

Conflict of Interest Statement for Tassinari
